# Rare nanoparticles shine colors with low-power STED

**DOI:** 10.1038/s41377-022-00863-z

**Published:** 2022-06-06

**Authors:** Xinzhu Xu, Peng Xi

**Affiliations:** College of Future Technology, Peking University, Beijing, China

**Keywords:** Nanoparticles, Nonlinear optics

## Abstract

The effect of cascade amplified depletion in lanthanide upconversion systems boosts their own emission bands inhibition, which facilitates multi-color nanoscopy with only one pair of low-power NIR CW lasers.

Stimulated emission depletion (STED) breaks the resolution limit of conventional optical microscopy through modulating the fluorescence spontaneous emission process. In conventional STED nanoscopy, the depletion wavelength is determined by the emission spectrum of the fluorochrome, so that the emission can be inhibited at the donut region surrounding the focal spot. Therefore, depletion wavelength selected by different dyes and the spatiotemporal coincidence of excitation and depletion are the most critical factors in STED. For achieving multicolor imaging, one needs to select different depletion wavelengths corresponding to different dyes, which may lead to a very high cost of the system. The practice of the commercial system is to use a white pulse laser as depletion source, and the specific dye corresponds to a specific filter combination, so that wavelength of the depletion is adjusted in real time to realize multicolor super-resolution imaging.

How can we achieve multicolor STED super-resolution with low-power illumination? Rare-earth-doped upconversion nanoparticles holds great promise. They are composed of a pair of sensitizer and emitter, because of the multi energy-level property of doped rare-earth elements, when the sensitizer is excited with a specific wavelength, the energy is transferred to the emitter, making the excited electrons of emitter to different excited energy levels, and these electrons occur cross-relaxation or transit back to the ground state, so generate multicolor fluorescence^1–4^. These emissions include both linear process and nonlinear processes (two-photon, three-photon, or higher-order photons). The difficulty of multicolor fluorescence is solved, but there is still a problem when applied to STED: the depletion is restricted to a certain energy level depending on the dye.

Previous work has achieved efficient population inversion at metastable high energy level of the emitter by selecting the co-doping of rare-earth elements and adjusting the doping concentration. When choosing depletion corresponding to the electron transition, low-power STED can be realized^5^; or a variety of rare-earth elements are doped as emitters, which realizes two-color super-resolution imaging^6^. However, in the above way, the depletion wavelength still depends on the energy level difference of the emitter. So is there a way to select a certain depletion to inhibit multicolor emission bands simultaneously?

To address this, Prof. Qiuqiang Zhan’s group from South China Normal University recently demonstrated a very brilliant emission depletion mechanism, namely stimulated-emission induced excitation depletion (STExD)^7^. Utilizing quasi-four-level Nd^3+^ as sensitizer and co-doping with different emitters in upconversion nanoparticles, it is found that under the 740 nm excitation, when the sensitizer is quenched with a NIR-II 1064 nm CW laser, the multichromatic emissions of a series of emitters which obtained energy from Nd^3+^ can be inhibited simultaneously. Importantly, the STExD process is characterized with an interesting effect of cascade amplified depletion, which can greatly reduce the saturation intensity. The depletion efficiency from the wavelength with respect to the multiphoton transition is much higher than to the one-photon transition under the same depletion power.

Figure 1a illustrates the principle of conventional STED: the wavelengths of the excitation and depletion lasers are carefully adapted with the various spectroscopic properties of multi-chromatic probes. Multiple pairs of laser beams have to be spatiotemporally overlapped with high-precision for multi-color imaging. Figure 1b is the principle of STExD: the same depletion which is only depended on the sensitizer itself. It can generate depopulation simultaneously for multiple emitting states of multi-chromatic probes by utilizing a single depletion wavelength to de-excite their common sensitizer. The reason why this cascade amplified depletion can improve the depletion efficiency from high-order photons is that, similar to the mechanism of multiphoton excitation process. Higher-order photons emissions exhibit stronger nonlinear dependence on the excitation power, intuitively, the depletion of sensitizer can be regarded as reducing excitation power, which has a greater effect on the higher-order ones (Fig. 2). Experimentally, the saturation intensity of the 450 nm (three-photon) emission was reduced to 23.8 kW cm^−2^, nearly three orders of magnitude lower than that of conventional STED dyes. Using the principle of STExD, the emission from other multi-photon transition progress can be simultaneously repressed under a single depletion, so as to achieve the purpose of simultaneous quenching of multiple colors. After that, the research group demonstrated high-efficiency STExD depletion in Nd/Yb/Er and Nd/Yb/Ho co-doped systems, which are also the first time to achieve the optical depletion of Ho^3+^-activated nanoparticles. In terms of imaging, STExD is utilized to image dual-color nanoparticles, and finally achieves 34 nm resolution. Meanwhile, the researchers achieved super-resolution imaging of the actin filaments in HeLa cell with spatial resolution <100 nm.

**Fig. 1:**
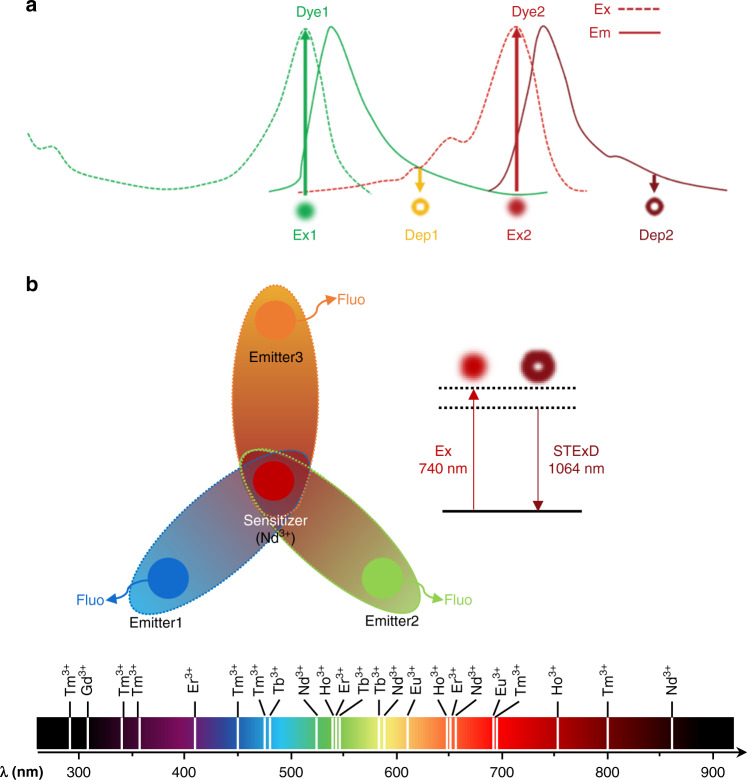
Schematic diagram of the processes. **a** The conventional STED, and **b** the STExD process

**Fig. 2:**
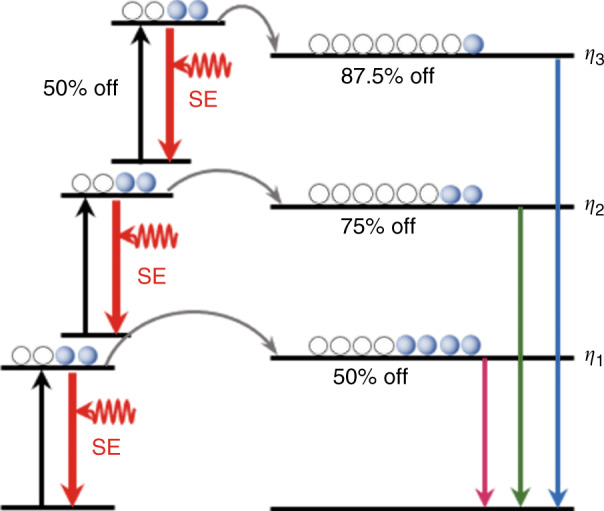
Illustration of cascade amplified depletion process

This work ingeniously takes advantage of the energy transfer and energy level structure between sensitizer-emitters of rare-earth doped upconverting nanoparticles, which provides conditions for directly inhibiting the excitation process. In turn, it can achieve high-efficiency suppression of multiphoton spontaneous emission. With Nd^3+^ as the sensitizer, multicolor super-resolution imaging can be achieved by using a single pair of CW excitation and depletion light under lower power, and depletion wavelength is only relied on the property of the sensitizer itself. Thus, the circumventing of limited photon-switchable emitters can also be achieved due to the intrinsic property of STExD^5,6^. Secondly, thanks to the merits of multiphoton effects in this UCNP system, the low-phototoxicity and deep-tissue imaging in vivo can be achieved^8,9^. Thirdly, this design idea may also provide extension using in normal down-conversion lanthanide-doped nanoparticles and other donor-acceptor nanosystems^10,11^ with expanding the bandwidth for multichannel super-resolution imaging. We believe this novel and versatile approach will pave new way for STED-based applications, for example, diffraction-unlimited imaging, sensing, optogenetics^2^, optical data storage, and lithography^12,13^.
